# COVID-19 Vaccination in Italian Children: The Limits of Parental Rights

**DOI:** 10.3390/children9050625

**Published:** 2022-04-27

**Authors:** Maricla Marrone, Benedetta Pia De Luca, Alessandra Stellacci, Luigi Buongiorno, Pierluigi Caricato, Gerardo Cazzato, Davide Ferorelli, Biagio Solarino, Pasquale Stefanizzi, Silvio Tafuri, Ettore Gorini, Michele di Landro, Alessandro Dell’Erba, Nicola Laforgia

**Affiliations:** 1 Section of Legal Medicine, Interdisciplinary Department of Medicine, University of Bari “Aldo Moro”, 70124 Bari, Italy; mariclamarrone@hotmail.it (M.M.); benedettapia.deluca@gmail.com (B.P.D.L.); alestellacci@gmail.com (A.S.); luigibuongiorno@gmail.com (L.B.); pierluigicaricato@libero.it (P.C.); davide.ferorelli@uniba.it (D.F.); biagio.solarino@uniba.it (B.S.); alessandro.dellerba@uniba.it (A.D.); 2 Section of Pathology, Department of Emergency and Organ Transplantation (DETO), University of Bari “Aldo Moro”, 70124 Bari, Italy; 3 Department of Biomedical Sciences and Human Oncology, University of Bari “Aldo Moro”, 70124 Bari, Italy; pasquale.stefanizzi@uniba.it (P.S.); silvio.tafuri@uniba.it (S.T.); 4 Department of Economics and Finance, University of Bari “Aldo Moro”, 70124 Bari, Italy; goriniettore@gmail.com (E.G.); michele.dilandro@policlinico.ba.it (M.d.L.); 5 U.O.C. Neonatology and NICU Policlinico Bari, University of Bari “Aldo Moro”, 70124 Bari, Italy; nicola.laforgia@uniba.it

**Keywords:** SARS-CoV-2, vaccine, social responsibility, children, public health, vaccination strategies, parental consent

## Abstract

SARS-CoV-2 vaccination campaigns initially targeted the adult population. After the authorization of the main agencies, including the EMA (European Medicines Agency), the European Vaccination Plan now involves young people between the ages of 12–17 and 5–11. In assessing the child’s “best interests”, the refusal of vaccination by parents or guardians, in addition to the increased circulation of the virus, is responsible for the risk of social distancing. This reduction in social contacts, particularly during very sensitive ages such as adolescence, has been linked to the increased incidence of psychiatric illness, a significant reason for extending vaccination against SARS-CoV-2 in these younger children. One may consider that government should issue a law that allows the child to decide on the vaccination plan, even without the consent of the parents or guardians, without the need for a judge’s ruling. The availability of the child should be the point of reference, according to the National Bioethics Committee, for consent to vaccination. The authors investigate the subject in depth in order to counteract vaccination hesitation, and promote the dissemination of correct scientific information, using every different possible communication tool, as well as social networks and schools.

## 1. Introduction

Although knowledge on the impact of the pandemic on mental and physical health is still limited and derives mainly from experiences that are only partially comparable to the current epidemic, for example those related to the SARS or Ebola epidemics [1,2], the demand for psychosocial interventions is likely to increase significantly in the coming months and years.

Since epidemics are unforeseen and unpredictable events of global significance, their history must inevitably be of help, tracing the path in which to set up the prevention, treatment, and protection measures for the health of the population.

The SARS-CoV-2 virus (severe acute respiratory syndrome coronavirus 2) is a viral strain of the coronavirus species, genetically related to the SARS virus, and belonging to the genus Betacoronavirus (family Coronaviridae). It causes COVID-19, “coronavirus disease-19”, which is a predominantly respiratory infectious disease, the first cases of which were found in China in December 2019.

The virus primarily affects the upper and lower respiratory tract, but can cause symptoms affecting all organs and systems [3]. In more than half of cases, the infection proceeds completely asymptomatically, and in around a third of the cases it presents flu-like symptoms (pauci-symptomatic form). In a minority of cases (about 5–6%), however, the disease can manifest itself in a moderate or severe form with the risk of developing complications, especially respiratory (respiratory failure, ARDS—acute respiratory distress syndrome).

From the beginning of the pandemic, that is, from December 2019 until January 2022, the deaths from COVID-19 in the world have been around 5.5 million, of which around 2 million occurred in Europe. In the various phases of the pandemic, the mortality rate has undergone some variations, progressively reducing, both in relation to the variants of the virus that have occurred in recent years, and in relation to the number of vaccinations administered.

To cope with the pandemic wave, alongside the targeted treatment of symptomatic forms, different types of vaccines have been tested and placed on the European market (Comirnaty, Pfizer; Spikevax, Moderna; Vaxzevria, Astrazeneca; Janssen, Johnson & Johnson).

Table 1 shows the main characteristics of the SARS-CoV-2 vaccines currently on the market in Italy.

There are additional vaccines used abroad, but are not yet approved by the EMA (European Medicines Agency), indicated in the following Table 2:

With specific reference to the Italian context, vaccination was initially intended only for the elderly and the occupational categories most exposed to the infection, thus excluding adults and children [18]. In particular, for mRNA vaccines, the first marketing authorization indicated a minimum age for administration of 16 (Comirnaty) and 18 (Spikevax) years.

In this regard, only a small number of minors with pathologies that weaken the immune system and/or the respiratory system (e.g., asthma, cystic fibrosis, type 1 diabetes mellitus, etc.), therefore more vulnerable to SARS-CoV-2 and developing serious illness, were vaccinated [5].

Following the greater availability of vaccine products and the modification of the marketing authorization by the regulatory bodies, with a view to contrasting the spread of the infection in children and reducing the circulation of the virus, the European Vaccination Plan has also involved the population aged 12–17 years since July 2021 and the age group 5–11 years since November 2021.

Unfortunately, the activity of anti-vaccine movements (anti-vax), also supported by the circulation of fake news on social networks [19], has prevented the vaccination of minors in family contexts in which one or both parents are “anti-vax”.

The consequent free movement of unvaccinated minors, often not attentive to the observance of the necessary safety measures (mask, hand washing, distance, etc.), difficult to “educate” in this sense, can make the preventive efforts ineffective in reducing the circulation of SARS-CoV-2.

On the contrary, the reduction of social contacts of minors careful to avoid contagion, especially in very delicate ages, such as adolescence, has been correlated with the increase in the incidence of psychiatric pathologies [20,21,22].

The authors therefore wanted to examine the situation relating to vaccination in minors in the Italian context.

Currently, in Italy, parents have to give consent to the vaccination of a minor aged between 12 and 17, for example by filling in a written consent form that the minor must bring to the vaccination appointment. If parental authority is shared, the consent of both parents or guardians is required.

Furthermore, in the judicial cases in which the conflict between parents (among whom a separation or divorce judgment was already pending) who expressed different addresses with respect to the vaccination of children, the orientation of the judge has always been in favor of vaccination, through the temporary suspension of the parental authority of the resistant parent.

In the absence of a specific rule, the Italian National Bioethics Committee unanimously expressed, on 29 July 2021, the opinion according to which minors between the ages of 12 and 17 can decide independently whether or not to undergo vaccination, regardless of the will of the parents and/or legal guardian. This intervention of the Bioethics Committee, although not having the force of law, is authoritatively inserted in the debate concerning the administration of vaccines to minors.

For this reason, it is essential to provide the minor with all the cognitive tools to facilitate adherence to the vaccination campaign, in the interest not only of his personal health, but of the health of the people close to him and, more generally, of the health community.

## 2. Aim and Scope

This article aims to evaluate the ethical and medico-legal implications of the administration of the COVID-19 vaccine in minors, starting from the Italian context.

Vaccination in minors currently requires the consent of both parents who may or may not be in favor of its administration. Any dissent on the part of one or both parents poses significant ethical challenges, mostly related to failure to achieve so-called herd or community immunity. Herd immunity is a mechanism that is established within a community, therefore if the vast majority of individuals are vaccinated, the circulation of an infectious agent is limited. In the view of a pandemic situation, it is of fundamental im-portance to achieve a high vaccination coverage for the good of the community, in order to reduce the spread of the virus and limit its contagion.

Therefore, the authors want to investigate in depth the importance to achieve a high vaccination coverage to combat vaccination hesitation and promote the dissemination of correct scientific information, in order to limit “anti-vax” movements, and increase vaccination coverage in terms of both the collective good and the best interests of the child.

## 3. Materials and Methods

A systematic review was elaborated following the preferred reporting items for systematic reviews and meta-analyses (PRISMA) guidelines.

The three different databases (PubMed, Google Scholar, and Web of Science) were consulted, using the main keywords “child vaccination” crossed with the terms “parental consent”, “COVID-19 vaccine”, “SARS-CoV-2”, “social distancing”, and/or “physical and mental health”. The research aimed to understand the reflections already posed on the topic.

The selection of the articles was carried out through the evaluation of both the title and the abstract.

The inclusion criteria were:

- Articles published in English;
- Type of paper: Original article, research article, systematic review, review.

The selected documents that met the inclusion criteria were then reviewed, as well as their references. Extensive research on the four COVID-19 vaccines approved in Europe and Italy (Pfizer-BioNTech, Moderna, Johnson & Johnson, and AstraZeneca) was also conducted on the websites of national and international drug regulatory authorities (FDA, EMA, AIFA).

To search for sentences, the Portal of Telematic Services (PST) of the Ministry of Justice was used, a tool that allows for the search and display of judgments on the merits only to REGINDE members, without the need for a subscription.

The most recent and most relevant Italian sentences on the subject of “vaccination of minors” have been selected.

## 4. Results

Among all the articles analyzed, the following types were found:

- Article: 11;
- Review: 5;
- Editorials: 4;
- Letter to the Editor: 4;
- Original article: 3;
- Rapid Communication: 3;
- Research paper: 2;
- Viewpoint: 1;
- Study protocol: 1.

**Figure 1:**
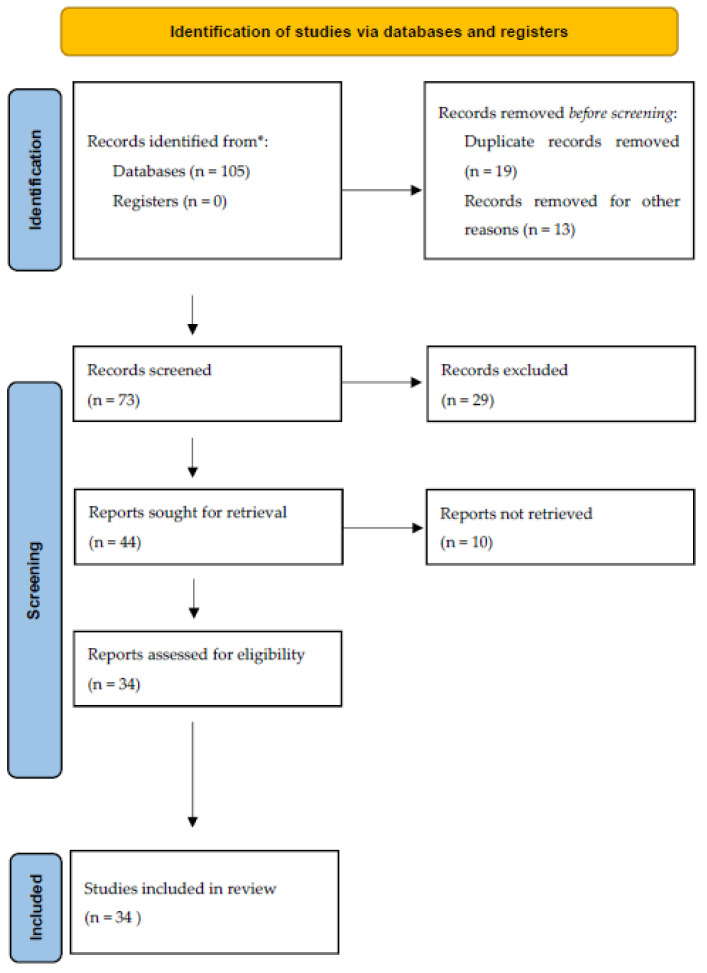
PRISMA guidelines used in review of literature.

Of the 34 isolated and analyzed articles, the majority (23 papers, 67.6%) came from European Union countries, while 1 article (2.9%) came from Taiwan, 1 article (2.9%) from Singapore, 4 articles (11.7%) from China, 1 article (2.9%) from Argentina, 1 article (2.9%) from Mexico, 1 article (2.9%) from Kazakhstan, 1 article from India, and 1 article from Israel (Figure 1).

## 5. Discussion

The COVID-19 pandemic and the necessary containment measures, including mainly physical distancing and isolation, are having detrimental consequences on the physical and mental health of the world’s population [23,24,25,26]. In fact, to reduce the spread of the virus, national and international bodies and institutions have set up tools such as case isolation, quarantine of contacts, and physical distancing in moments of sociality almost everywhere in the world. However, the psychological reactions that develop following the quarantine, such as frustration, loneliness and worries about the future, are the most common reactions and represent well-known risk factors for several mental disorders, including anxiety, affective and anxiety disorders, and post-traumatic stress [27,28,29]. In the era of lockdown or in any case in the phases in which the rules on physical distancing are more stringent, the Internet and social media networks can be useful for reducing isolation and increasing the opportunities to stay in contact with family, friends, and colleagues at all times [30,31]; however, they can also represent risk factors for the development of mental disorders, such as gambling addictions. Furthermore, the networks can also have a negative impact on the mental health of the most vulnerable people, such as minors, as it disseminates an uncontrolled amount of information.

In contrast, no significant adverse effects were found in a retrospective study that included over 1 million adults and 600 children aged 12 to 16 who received the vaccine [32].

In Europe, as of 28 May 2021 (Circular EMA/289461/2021), EMA’s Committee for Medicinal Products for Human Use (CHMP) has recommended granting an extension of indication for Comirnaty, an COVID-19 vaccine, to include use in children 12 to 15 years of age; the vaccine has already been approved for adults and adolescents from 16 years of age.

The effects of Comirnaty in the pediatric population were studied in 2260 children aged 12 to 15 years. This study was conducted in accordance with the Comirnaty Pediatric Investigation Plan (PIP), approved by the EMA’s Pediatric Committee (PDCO) [33].

The study showed that the immune response (antibody levels against SARS-CoV-2) induced by Comirnaty in this age group was comparable to that seen in the 16–25 year age group, with 100% efficacy in disease prevention (although the actual rate can be between 75% and 100%).

The most common side effects seen in children aged 12 to 15 years are similar to those seen in adults: pain at the injection site, fatigue, headache, pain in muscles and joints, and chills and fever. These effects are usually mild or moderate in severity, and resolve within a few days of vaccination.

Therefore, the CHMP concluded that the benefits of Comirnaty in this age group outweigh its risks.

It is known that COVID-19 affects children much less severely than adults, typically with flu-like symptoms; however, starting from June 2021, there has been an increase in the incidence of infections and diseases among children. In addition, around 1 in 3 children admitted to hospital require intensive care.

Furthermore, minors >12 years of age appear to be “more at risk” than younger ones, and show a higher mortality rate [33].

New studies have shown that when children develop COVID-19 infection, the duration of the symptoms and the illness last for a longer period of time [34].

The potential role of children as factors of infection should also be considered, since social settings such as schools have long been known as potential amplifiers of epidemic diseases, particularly respiratory diseases. In this context, the role of the school in the amplification of influenza epidemics has been extensively studied.

From all of the above, it is quite clear that it is riskier not to vaccinate children, rather than subject them to the very rare adverse effects, however modest, of vaccination.

Vaccination of minors thus becomes a problem that must be addressed by the whole of society. Currently, the National Bioethics Committee has unanimously expressed, on 29 July 2021, its opinion, according to which minors between the ages of 12 and 17 can, independently, decide whether or not to undergo vaccination, regardless of the will of the parents and/or legal guardian. The society must therefore deal with two types of situations that may arise:

- In the first place, there is the case in which the parents or a legal guardian belonging to “anti-vax” movements or simply opposed to vaccination due to the prevalence of feelings of fear and anguish, do not want to vaccinate the minor, who instead wants to undergo vaccination;
- There is also the case in which the minor expresses the will not to be vaccinated, and the parents or legal guardian, on the other hand, want it to be carried out.

Obviously, if it is decided that it is correct to accept that the minor’s opinion must have absolute validity, this must be the case both in the case of willingness to undergo the vaccination and vice versa. This presupposes that the child must be adequately informed about the benefits and risks of vaccination in order to be able to make decisions in their own interests, as well as in the interests of those around them.

In recent months, the Italian Judicial Authority has intervened, ruling on the limitation of parental responsibility, affirming the denial, based on irrational ideological prejudices, of consent to the administration of the vaccine, as it is harmful to the health of children.

Worthy of note are the very recent rulings of the Italian Court, which, in the face of the refusal of one of the two parents of the minor, in the interest of the latter and with the support of the other parent, favor vaccination (Table 3).

The judge, in each of these cases, took into account Art. 3 of Law no. 219 of 2017 (Italian law defining informed consent in the health sector, as well as with regard to living wills), according to which the minor has the right to strengthen his or her ability to understand and decide. Therefore, the informed consent to the minor’s medical treatment is expressed or refused by the exercisers of parental authority or by the guardian taking into account the will of the minor, in relation to his age and degree of maturity, and having as its main purpose the protection of health, psychophysics, and the life of the minor in full respect of their dignity.

## 6. Conclusions

The question is still open at the moment, and will continue to be open for the foreseeable future.

The attempt proposed by the authors is aimed at underlining the central role played by health and non-health information provided to both minors and parents. The general practitioner and/or pediatrician, as well as the family socio-cultural context, thus acquire an important value for the purposes of a correct framing of the problem, which in turn is important for orienting subsequent choices.

Furthermore, in this regard, the mass media have the potential to reach a large number of people and, therefore, represent a useful means of disseminating accurate scientific information on COVID-19 disease and vaccination.

The network also has the potential to reach large numbers of people at relatively low cost, with important implications for its role in science communication on topics, such as adolescent vaccination. However, it is essential to know how to distinguish the right information from “fake news”, which contributes to the creation of unjustified alarms about vaccination.

It would therefore be desirable that, even in the field of media journalism, there would be a “filter” with scientific expertise on the subject that favors correct information, preventing the dissemination of notions without scientific basis.

It would also be useful to increase the skills of school staff, in particular of teachers of scientific disciplines, so that they can provide technical notions that favor the understanding of the problem in all its aspects in minors. The compulsory short Master’s degree for science teachers could create the basis for correct information and education. All of this is intended to reduce vaccination hesitations and facilitate the administration of the vaccine for COVID-19 in order to slow down the circulation of the viral agent, with a view to the collective good.

A last thoughtful note recognizes the ability of the minor, especially if they are a teenager, to inform themselves correctly and to correctly evaluate the information acquired on the subject.

Specific tools are needed for assessing the knowledge of minors when it comes to being vaccinated or not, as well as assessing whether this knowledge is sufficient for their decision to be correct and well-informed. This last aspect should be dealt with by child neuropsychiatrists, specialists in the sector.

## Figures and Tables

**Table 1:** Main characteristics of the SARS-CoV-2 vaccines currently on the market in Italy [4,5,6,7].

	Comirnaty (BioNTech/Pfizer)	Spikevax (Moderna)	Vaxzevria (Formerly AstraZeneca)	Janssen (Johnson & Johnson)
Minimum age	≥12 years		≥18 years	
Method of administration	2 administrations at least 3 weeks apart	2 administrations at least 4 weeks apart	2 administrations at 4/12 weeks apart	Single administration
Mechanism of action	Molecule of messenger RNA, contained in lipid vesicles, which fuse with human cells		Recombinant vector, based on chimpanzee adenovirus (ChAdOx1)	Recombinant vector, based on type 26 human adenovirus
	→ Coding of the spike glycoprotein (S) of SARS-CoV-2, which induces, in the host, an immune response against the SARS-CoV-2 virus.			

**Table 2:** Some of the SARS-CoV-2 vaccines used abroad but not approved by the EMA [8,9,10,11,12,13,14,15,16,17].

Vaccine	Producer	Active Principle
Sputnik V	Gameleya Research Institute of Epidemiology (Russia)	Viral Vector
Sputnik V Light		
Convidencia (Ad5-nCoV)	CanSino Biologics (China)	
BBIBP-CorV	Sinopharm (China)	Inactive virus
CoronaVac	Sinovac (China)	
Covaxin (BBV152)	Bharat Biotech + Indian Councid of Medical Research	
QazVac	Research Institute for Biological Safety Problems in Kazakhstan	
IMBCAMS COVID-19 vaccine	Institute of Medical Biology of the Chinese Academy of Medical Sciences (IMBCAMS)	
COVID-19 Inactivated Vaccin (COVIran Barekat)	Shifa Pharmed Industrial Group in Iran	
EpiVacCorona	VECTOR center of Virology (Russia)	Peptide subunit
RBD-dimer (ZF2001)	Anhui Zhifei Longcom (Cina)	
Abdala	Center for Genetic Engineering and Biotechnology in Cuba	

**Table 3:** Rulings of the Italian Court on the matter in question.

**Court of Milan ^1^**
Topic → Refusal of the mother to have her 11-year-old daughter undergo the vaccine, molecular swabs for the diagnosis of COVID-19 and antigen tests, as well as the use of a mask, which is considered “harmful”.Outcome → Decree of 2–13 September 2021 (procedure inscribed under n.ro 6014/2021 R.G.) which authorizes the father to provide autonomously, without the consent of the mother, thus limiting the maternal parental responsibility in relation to all issues related to mandatory or optional vaccinations, swabs, the possible administration of the COVID-19 vaccine, and the use of the mask, attributing it exclusively to the father.
**Court of MONZA ^2^**
Theme → Unjustified refusal opposed by the father; willingness in favor of vaccination expressed by the 15-year-old and 6-month-old child.Outcome → Decree of 22 July 2021 which authorized the administration of the COVID-19 vaccine to the minor, giving the mother the right to accompany the child to a vaccination center and sign the relative informed consent, even in the absence the consent of the other parent.
**Court of BRINDISI**
Topic → Father is anti-vax for people younger than 14 years old due to being “fragile”Outcome → Authorized administration as “in vaccination, since it is impossible to seek zero risk, a risk-benefit assessment must be carried out”; in this specific case, the benefits associated with the administration of the COVID-19 vaccine outweighed the risks associated with the vaccination itself.

^1^ Decree of 2–13 September 2021 of the Court of Milan: “The father has been authorized to independently provide, without the mother’s consent, to carry out the optional vaccinations recommended by art. 1, paragraph 1-quater of the D.L. n. 73/2017, converted with amendments by law 219/2017 and provided for by the LEA to be administered to the minor daughter at the age of 12, according to the indications of the doctor-pediatrician of reference. The father authorized to have his daughter carry out the “anti-Covid” swab (in the forms of molecular test, rapid antigen test, traditional or rapid serological test, salivary test as needed) without the mother’s consent, as often as necessary of the case). The father has been authorized to independently assess, without the mother’s agreement, whether it is necessary or even only appropriate to administer the COVID-19 vaccine to the younger daughter, providing accordingly. Arranged that the daughter uses the mask necessary to limit the possibility of contagion from COVID-19 in all situations imposed by law or in any case in the event of a gathering, delegating the father to make sure that this happens. Maternal parental responsibility is limited in relation to all issues related to mandatory or optional vaccinations, swabs, the possible administration of the COVID-19 vaccine and the use of the mask, attributing it exclusively to the father”. ^2^ Decree of 22 July 2021 of the Court of Milan: “[...] In other words, in evaluating the options supported respectively by the mother and the father, the Judge must take into account the existence of serious damage to health and the spread of the disease on the national territory. In the same way as these criteria, decisions in the negative sense were taken where the vaccine concerned pathologies with little diffusion in our country, circumstances that do not occur in the case of COVID-19, a pathology that notoriously in a significant number of cases has had serious consequences and / or fatal with a very wide spread not only on the national territory, but worldwide, with very serious effects on the health systems of many countries [...] As for the efficacy of the vaccine in preventing the disease and in contrasting the spread of the infection, both the national scientific community that internationally, on the basis of continuously updated studies, agrees that the vaccines approved by national and international regulatory authorities are highly effective in protecting both individuals and the community from serious illness and in particular vulnerable subjects with a risky relationship. benefits where the benefits outweigh the risks in all ranges of age, including those younger that they are, even those in which the circulation of the virus is higher due to greater socialization. The wide vaccination coverage then allows to slow down and control the transmission of the disease with beneficial effects for the whole community. On the contrary, the absence of vaccination coverage, especially in the presence of increasingly contagious variants, entails, on the one hand, a greater risk for individuals, including minors, of contracting the disease and, on the other, negative repercussions on the social and working life of people and, as regards minors, on their educational path, limiting the possibility of access to training facilities [...]”.

## Data Availability

Not applicable.
